# Supplementary material for: 2,4-dienoyl-CoA reductase regulates lipid homeostasis in treatment-resistant prostate cancer
Source: Nat Commun. 2020 May 19;11:2508. doi: 10.1038/s41467-020-16126-7 (PMC7237503; doi:10.1038/s41467-020-16126-7)
Supplement: Supplementary file 1 — Supplementary Information [file 41467_2020_16126_MOESM1_ESM.pdf]

Supplementary Information

2,4-dienoyl-CoA reductase regulates lipid homeostasis in treatment-resistant prostate cancer

Blomme et al.

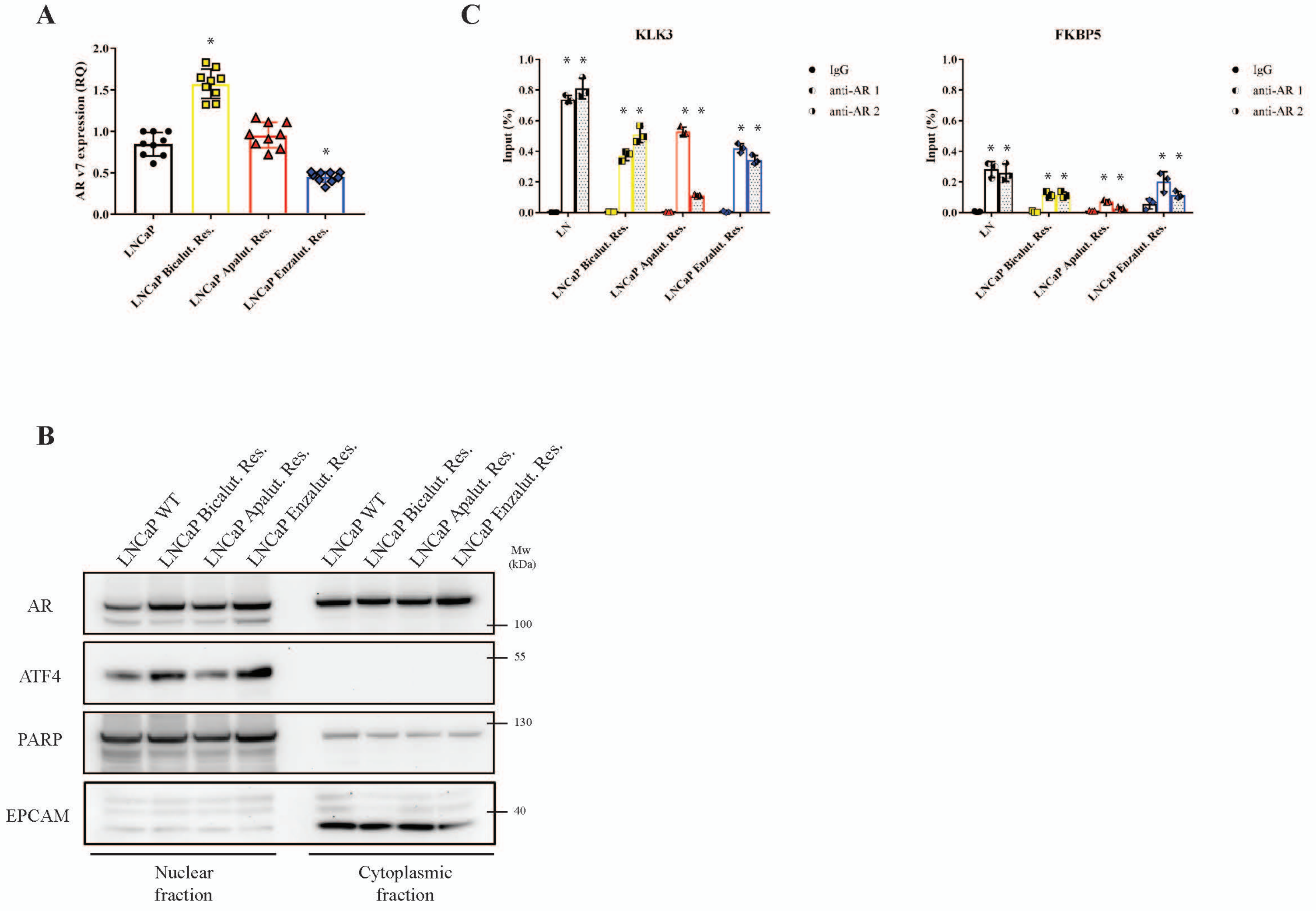

Supplementary Figure 1

Supplementary Figure 1: AR signalling is conserved in ARI-resistant cells. A, RT-qPCR analysis of *ARv7* expression in WT and ARI-resistant LNCaP cells. *CASC3* is used as a normalising control. **B,** Western blot analysis of AR expression on nuclear and cytoplasmic extracts of WT and ARI-resistant LNCaP cells. ATF4 and PARP are used as nuclear markers, EPCAM is used as a marker of the non-nuclear fraction. **C**, RT-qPCR analysis of the *KLK3* and *FKBP5* promoters after anti-AR chromatin immunoprecipitation performed in WT and ARI-resistant LNCaP cells. Panel **A:** n = 9 (3 independent biological experiments performed in triplicates). Panel **B:** representative image from 2 independent biological experiments. Panel **C:** n = 3 independent biological experiments. Panels **A, C**: Data are presented as mean values +/- SD. Panels **A, C**: *p-value < 0.05 using a 1-way ANOVA with a Dunnett's multiple comparisons test. Source data are provided as a Source Data File.

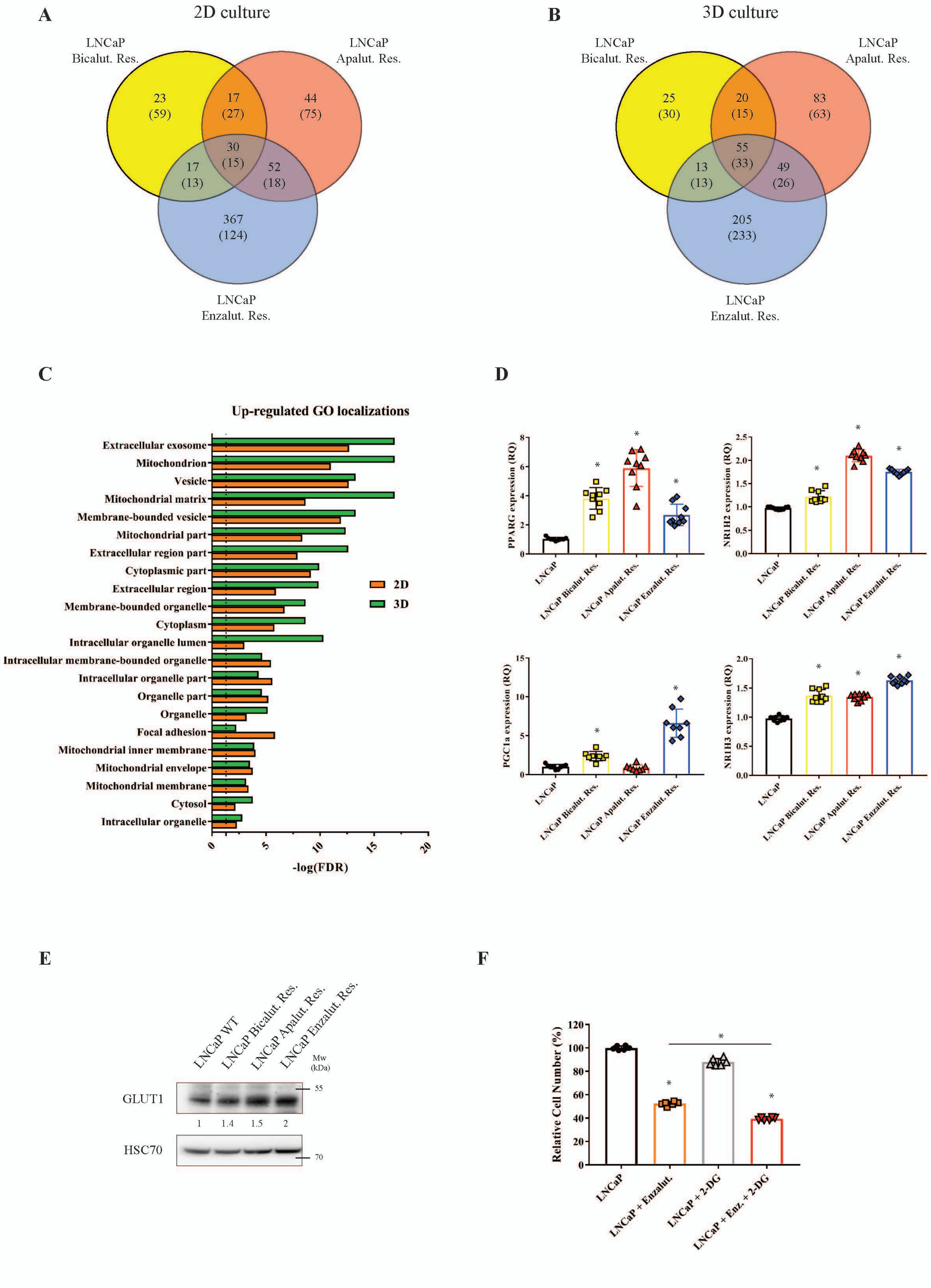

Supplementary Figure 2

Supplementary Figure 2: Omics analysis of ARI-resistant cells reveal altered glucose metabolism. A-B, Venn diagrams highlighting proteins that are significantly modulated (p-value < 0.05, FC > 1.5) in ARI-resistant cells when compared to WT LNCaP, identified in 2D (**A**) or 3D (**B**) comparisons. Down-regulated proteins are indicated in brackets. **C,** Enriched GO localisations up-regulated in the proteomic analysis of ARI-resistant cells when compared to WT LNCaP. Selected proteins were significantly modulated in at least 2 out of 3 conditions. Cellular localisation enrichment analysis was performed using the STRING database (http://string-db.org). **D**, RT-qPCR analysis of *PPARG*, *PGC1α, LXRα (NR1H3) and LXRβ (NR1H2)* expression in WT and ARI-resistant LNCaP cells. *CASC3* is used as a normalising control. **E**, Western blot analysis of GLUT1 expression in WT and ARI-resistant LNCaP cells. HSC70 is used as a sample loading control. **F,** Cell proliferation of WT LNCaP cells treated for 48 hours with enzalutamide (5 µM) and 2-deoxy-D-glucose (250 µM). Panel **D:** n = 9 (3 independent biological experiments performed in triplicates). Panel **E:** representative image from 3 independent biological experiments. Panel **F:** n = 6 (3 independent biological experiments performed in duplicates). Panels **D, F**: Data are presented as mean values +/- SD. Panels **D, F**: *p-value < 0.05 using a 1-way ANOVA with a Dunnett's multiple comparisons test. Source data are provided as a Source Data File.

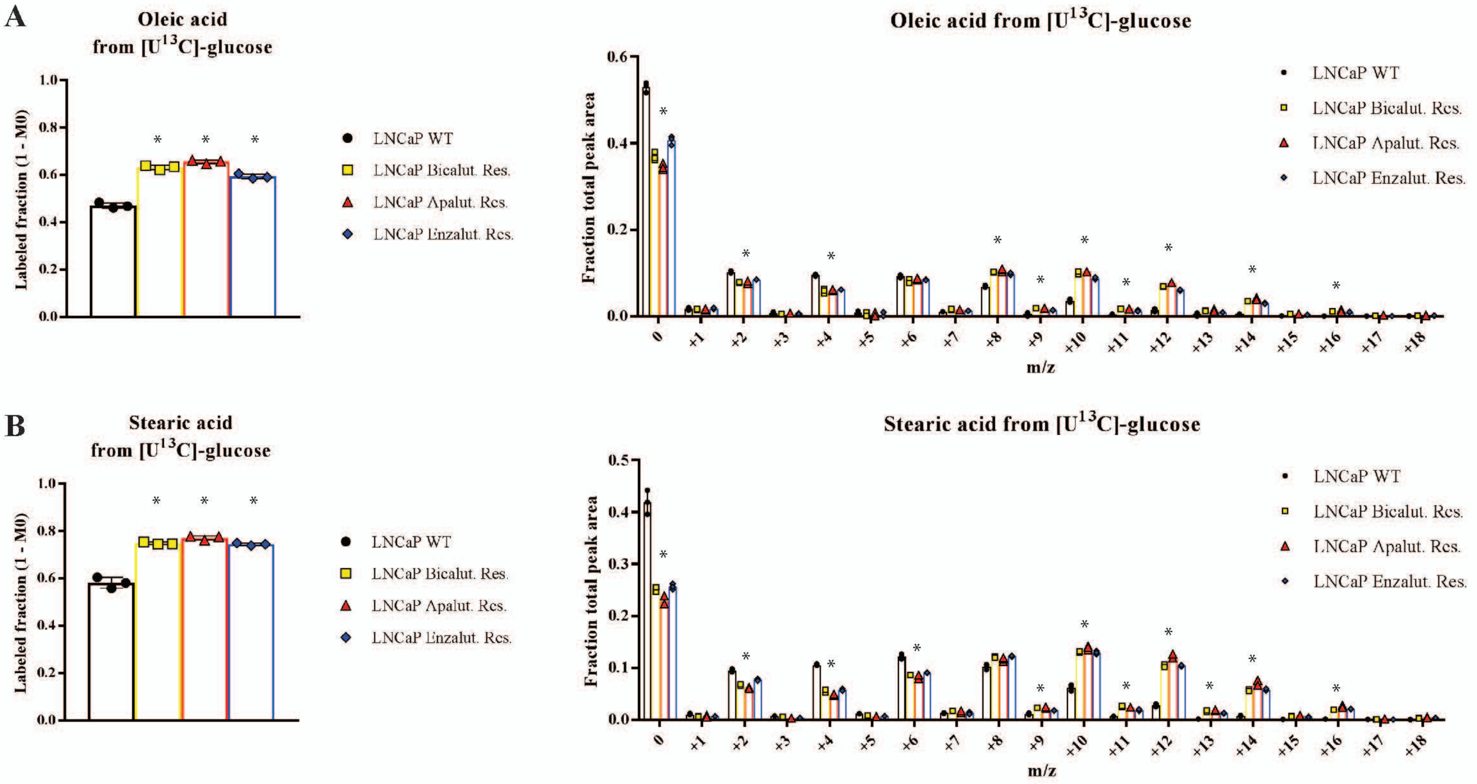

Supplementary Figure 3

Supplementary Figure 3: Lipid metabolism is strongly dysregulated in ARI-resistant cells. A-B, Labelled oleate (A) and stearate (B) fractions derived from ^{13}C-glucose (left panel) and relative isotopologue distribution of oleic and stearic acid in ARI-resistant and WT LNCaP cells following ^{13}C-glucose incubation for 72 hours (right panel). Panel **A, B**: n = 3 independent wells from the same cell culture. Panel **A, B**: Data are presented as mean values +/- SD. Panel **A, B**: *p-value < 0.05 using a 1-way ANOVA with a Dunnett's multiple comparisons test. Source data are provided as a Source Data File.

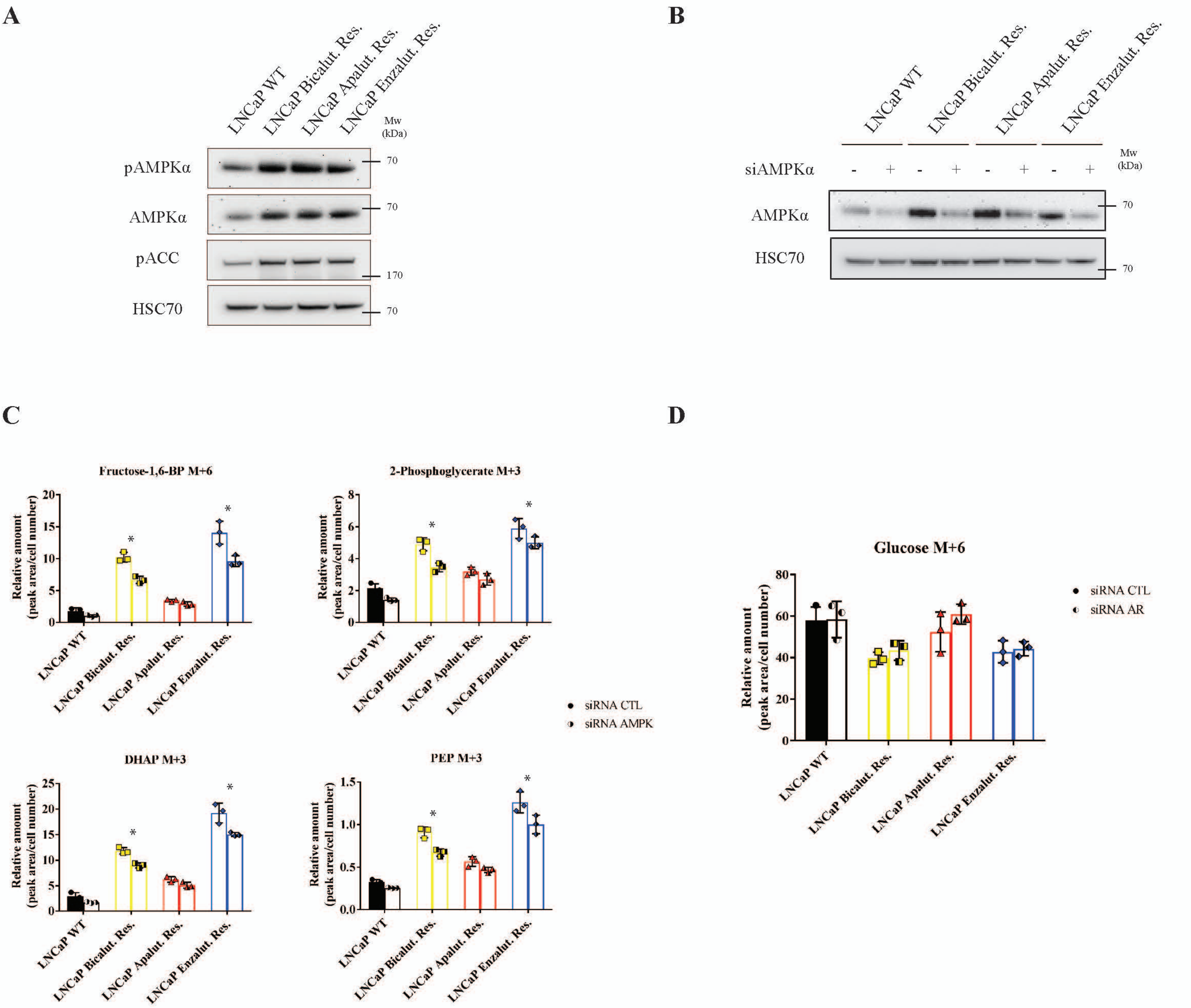

Supplementary Figure 4

Supplementary Figure 4: Increased glucose metabolism in ARI-resistant cells partially relies on AMPKα signalling. **A**, Western blot analysis of AMPKα, phospho-AMPKα and phospho-ACC expression in WT and ARI-resistant LNCaP cells. HSC70 is used as a sample loading control. **B**, Western blot analysis of AMPKα expression in WT and ARI-resistant LNCaP cells following AMPKα siRNA silencing. HSC70 is used as a sample loading control. **C**, Expression of labelled isotopologue of selected metabolites in ARI-resistant and WT LNCaP cells with suppressed AMPKα expression and following 1 hour ^{13}C-glucose incorporation. **D**, Expression of labelled isotopologue of m+6 glucose in ARI-resistant and WT LNCaP cells silenced for AR and following 1 hour ^{13}C-glucose incorporation. Panels **A, B:** representative image from 3 independent biological experiments. Panels **C, D**: n = 3 independent wells from the same cell culture. Panels **C, D**: Data are presented as mean values +/- SD. Panel **C**: *p-value of labelled fraction < 0.05 using a 2-way ANOVA with a Sidak’s multiple comparisons test. DHAP: dihydroxyacetone-phosphate; PEP: phosphoenolpyruvate. Source data are provided as a Source Data File.

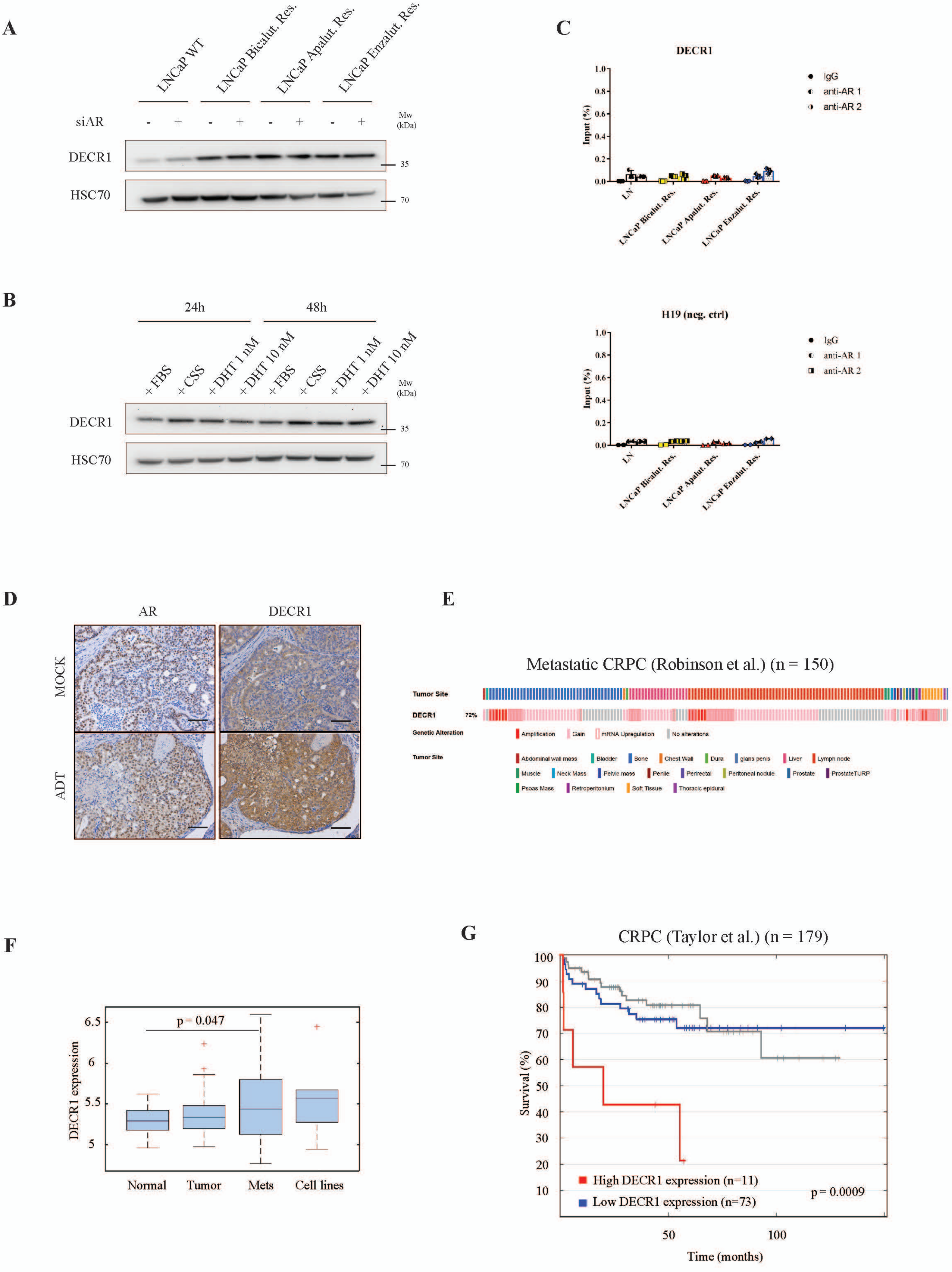

Supplementary Figure 5

Supplementary Figure 5: DECR1 is a potential target for CRPC. **A**, Western blot analysis of DECR1 expression in WT and ARI-resistant LNCaP cells following AR siRNA silencing. HSC70 is used as a sample loading control. **B**, Western blot analysis of DECR1 expression in WT LNCaP cells treated with DHT in androgen-deprived condition (CSS). HSC70 is used as a sample loading control. **C**, RT-qPCR analysis of the *DECR1* and *H19* promoters after anti-AR chromatin immunoprecipitation performed in WT and ARI-resistant LNCaP cells. *H19* is used as a negative control. **D**, Immunohistochemical expression of AR and DECR1 in prostate sections from *Nkx3.1 Pten*$^{fl+/-}$ *Spry2*$^{fl+/-}$ mice. Scale bars represent 100 µm. **E**, Percentage of metastastic CRPC lesions showing genomic (copy number gain or amplification) or mRNA alteration (z-score >= 1.5) for DECR1 using the GSE5988 dataset (n=150). **F**, Gene expression analysis of DECR1 in normal and tumoural prostate tissues according to the GSE21034 dataset (n=179). Center line corresponds to median of data, top and bottom of box correspond to 75th and 25th percentile, respectively. Whiskers extend to adjacent values (minimum and maximum data points not considered outliers). **G**, Kaplan-Meier survival analysis of prostate cancer patients divided according to high (red), medium (grey) and low (blue) levels of DECR1 expression using the dataset from Robinson et al. doi:10.1016/j.cell.2015.06.053. Panels **A, B:** representative image from 2 independent biological experiments. Panel **C**: n = 3 (DECR1) and 2 (H19) independent biological experiments. Panel **D**: n = 3 mice per condition. Panel **C**: Data are presented as mean values +/- SD. Panel **C**: *p-value < 0.05 using a 1-way ANOVA with a Dunnett's multiple comparisons test. Panel **F**: statistical analysis was performed using a pairwise ANOVA. Panel **G**: statistical analysis was performed using a logrank test. Source data are provided as a Source Data File.

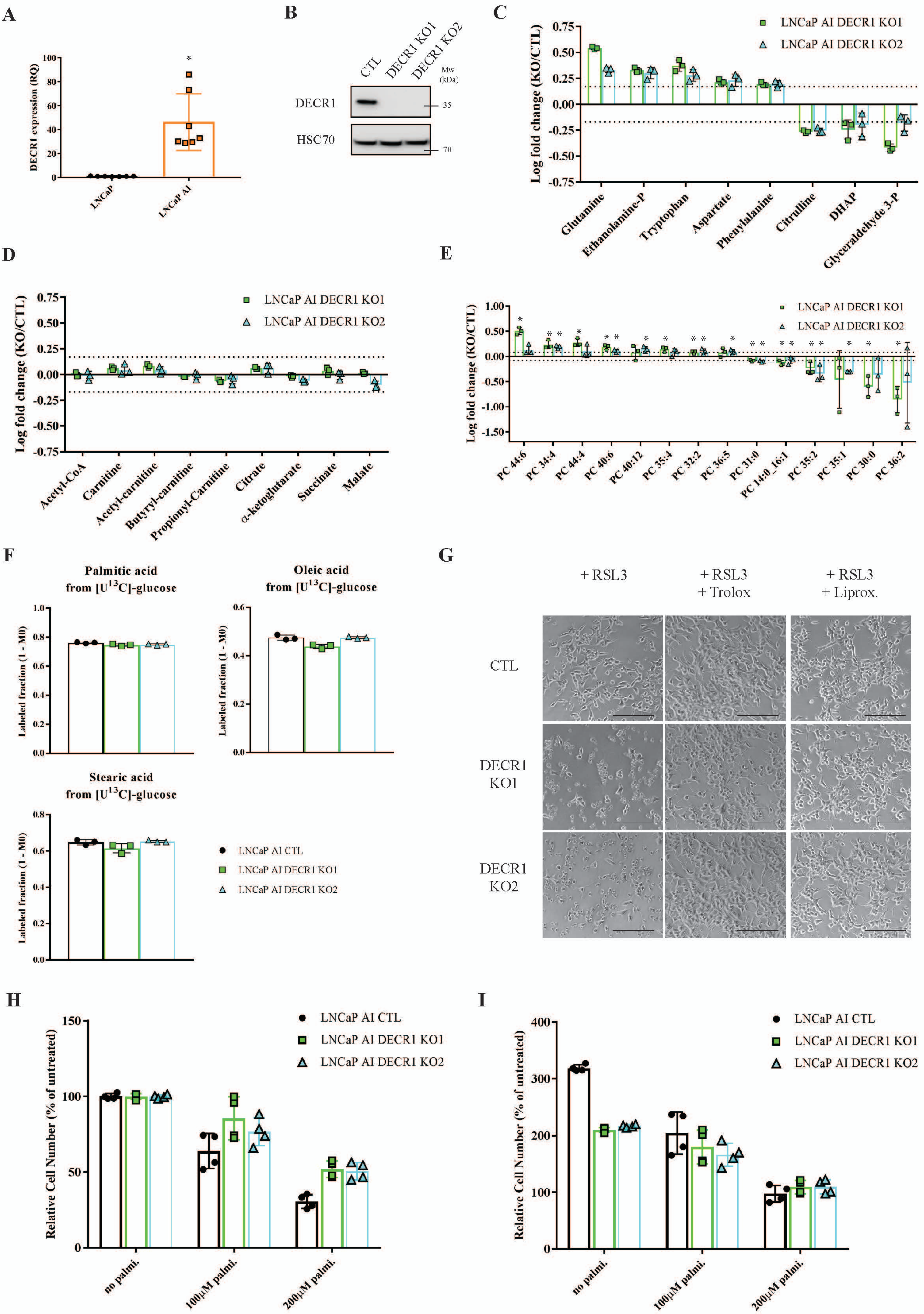

Supplementary Figure 6

Supplementary Figure 6: DECR1 loss alters lipid homeostasis and sensitises CRPC cells to ferroptosis. A, RT-qPCR analysis of *DECR1* expression in LNCaP and LNCaP AI cells. *CASC3* is used as a normalising control. **B**, Western blot analysis of DECR1 expression in DECR1 KO cells. HSC70 is used as a sample loading control. **C,** Steady-state levels of significantly regulated metabolites in DECR1 KO cells compared to CTL cells (FC > 1.5, $p < 0.05$ using a 1-way ANOVA with a Dunnett's multiple comparisons test). **D**, Steady-state levels of Acetyl-CoA, TCA cycle and carnitine derivatives in DECR1 KO cells compared to CTL cells. **E**, Steady-state levels of significantly altered phosphatidylcholines (PC) in DECR1 KO cells when compared to CTL cells (FC > 1.2). Selected lipids were significantly altered in at least one of the two KO cells (*$p < 0.05$ using two-sided Student's t-test). **F**, Labelled palmitate, oleate and stearate fractions derived from ^{13}C-glucose in CTL and DECR1 KO cells following ^{13}C-glucose incubation for 72 hours. **G,** Representative pictures of DECR1 KO and CTL cells treated for 48 hours with RSL3 (10 µM) and Trolox (20 µM) or Liproxstatin (50 nM). Scale bar represents 100 µm. **H**, Cell proliferation of DECR1 KO and CTL cells treated for 72 hours with palmitic acid. Cell count is normalised to initial number of cells at the start of the experiment (T0) and expressed as a relative percentage of untreated cells for each condition. **I**, same as **H** but cell count is normalised to initial number of cells at the start of the experiment (T0). Panel **A**: n = 7 independent biological experiments. Panels **B, G:** representative image from 3 independent biological experiments. Panels **C, D, E**: n = 3 independent biological experiments. Panel **F**: n = 3 independent wells from the same cell culture. Panels **H, I**: n = 4 (2 independent biological experiments performed in duplicates). Panels **A, C, D, E, F, H, I**: Data are presented as mean values +/- SD. Panel **A**: *p-value < 0.05 using two-sided Student's t-test. DHAP: dihyroxyacetone-phosphate. PC: phosphatidylcholine. Source data are provided as a Source Data File.

Supplementary Table 1: List of primers used in this study.

Target Gene	Forward	Reverse
AR-FL	aagagaagtacctgtgcgcc	ttcagattaccaagtttcttcag
AR-V7	aagagaagtacctgtgcgcc	tcagggtctggtcattttga
CASC3	ggggttccagttaatacaagtttc	gccagctgtatttctcttctgag
DECR1	gcttcttggattaatggagca	tcgttgaattcccctgaaat
FKBP5	ggatatacgccaacatgttcaa	ccattgctttattggcctct
KLK3	gtgcttgtggcctctcgt	cagcaagatcacgcttttgt
PPARG	aaaatcaagttcaaacacatcacc	cctgaaagatgcggatgg
PGC1A	tgaaccaattttaaatacattctcctt	accaagtattccactcatgtcaac
NR1H2	ttgaaggacttcacctacagca	actcgaagatggggttgatg
NR1H3	gttataaccgggaagactttgc	aaactcggcatcattgagttg

Supplementary Table 2: List of primary antibodies used in this study.

Protein	Reference	Company
PSA	sc-7638	Santa Cruz Biotechnology
AR	sc-816	Santa Cruz Biotechnology
HSC70	sc-7298	Santa Cruz Biotechnology
FKBP5	#12210	Cell Signaling Technology
ACC	#3676	Cell Signaling Technology
ACLY	#4332	Cell Signaling Technology
p-ACLY	#4331	Cell Signaling Technology
PKM1/2	#3190	Cell Signaling Technology
GLUT1	#12939	Cell Signaling Technology
AMPKa	#2603	Cell Signaling Technology
p-AMPKa	#2535	Cell Signaling Technology
CPT1a	#12252	Cell Signaling Technology
BIP	#3177	Cell Signaling Technology
DNAJC3	#2940	Cell Signaling Technology
XBP1s	#40435	Cell Signaling Technology
CHOP	#2895	Cell Signaling Technology
EPCAM	#93790	Cell Signaling Technology
ATF4	#11815	Cell Signaling Technology
PARP	#9532	Cell Signaling Technology
DECR1	ab95965	Abcam
GPX4	ab125066	Abcam
