# Supplementary material for: 2,4-dienoyl-CoA reductase regulates lipid homeostasis in treatment-resistant prostate cancer
Source: Nat Commun. 2020 May 19;11:2508. doi: 10.1038/s41467-020-16126-7 (PMC7237503; doi:10.1038/s41467-020-16126-7)
Supplement: Supplementary file 3 — Description of Additional Supplementary Files [file 41467_2020_16126_MOESM3_ESM.pdf]

Description of Additional Supplementary Files

File Name: Supplementary Data 1
Description: Modulated proteins (FC = 1.5, $p < 0.05$) in BIC, APA, ENZ compared to LNCaP.

File Name: Supplementary Data 2
Description: Raw data for LC-MS lipidomics.
