# Supplementary material for: 2,4-dienoyl-CoA reductase regulates lipid homeostasis in treatment-resistant prostate cancer
Source: Nat Commun. 2020 May 19;11:2508. doi: 10.1038/s41467-020-16126-7 (PMC7237503; doi:10.1038/s41467-020-16126-7)
Supplement: Supplementary file 6 — Reporting Summary [file 41467_2020_16126_MOESM6_ESM.pdf]

nature research

Corresponding author(s): Hing Y. Leung

Last updated by author(s): Jan 12, 2020

Reporting Summary

Nature Research wishes to improve the reproducibility of the work that we publish. This form provides structure for consistency and transparency in reporting. For further information on Nature Research policies, see Authors & Referees and the Editorial Policy Checklist.

Statistics

For all statistical analyses, confirm that the following items are present in the figure legend, table legend, main text, or Methods section.

n/a	Confirmed	
☐	☒	The exact sample size (*n*) for each experimental group/condition, given as a discrete number and unit of measurement
☐	☒	A statement on whether measurements were taken from distinct samples or whether the same sample was measured repeatedly
☐	☒	The statistical test(s) used AND whether they are one- or two-sided<br>*Only common tests should be described solely by name; describe more complex techniques in the Methods section.*
☒	☐	A description of all covariates tested
☐	☒	A description of any assumptions or corrections, such as tests of normality and adjustment for multiple comparisons
☐	☒	A full description of the statistical parameters including central tendency (e.g. means) or other basic estimates (e.g. regression coefficient) AND variation (e.g. standard deviation) or associated estimates of uncertainty (e.g. confidence intervals)
☐	☒	For null hypothesis testing, the test statistic (e.g. *F*, *t*, *r*) with confidence intervals, effect sizes, degrees of freedom and *P* value noted<br>*Give P values as exact values whenever suitable.*
☒	☐	For Bayesian analysis, information on the choice of priors and Markov chain Monte Carlo settings
☒	☐	For hierarchical and complex designs, identification of the appropriate level for tests and full reporting of outcomes
☒	☐	Estimates of effect sizes (e.g. Cohen's *d*, Pearson's *r*), indicating how they were calculated

Our web collection on statistics for biologists contains articles on many of the points above.

Software and code

Policy information about availability of computer code

Data collection	Patient RNAseq data were publicly available.<br>For omics analysis, the following softwares were used:<br>Maxquant v1.5.5.1<br>Thermo Xcalibur v4.2.47<br>Compound Discoverer v3.0
Data analysis	Gene expression data were plotted using Matlab.<br>For omics analysis, the following softwares were used:<br>Perseus v1.5.5.3<br>Tracefinder v4.0<br>Lipidex v3.0<br>Mass Hunter B.06.00<br>Graphs and Heatmaps were generated using Graphpad PRISM v7.05 and R v36.1

For manuscripts utilizing custom algorithms or software that are central to the research but not yet described in published literature, software must be made available to editors/reviewers. We strongly encourage code deposition in a community repository (e.g. GitHub). See the Nature Research guidelines for submitting code & software for further information.

Data

Policy information about availability of data

All manuscripts must include a data availability statement. This statement should provide the following information, where applicable:

- Accession codes, unique identifiers, or web links for publicly available datasets
- A list of figures that have associated raw data
- A description of any restrictions on data availability

The raw files and the MaxQuant search results files have been deposited as partial submission to the ProteomeXchange Consortium via the PRIDE partner

repository [Perez-Riverol Y, Csordas A, Bai J, Bernal-Llinares M, Hewapathirana S, Kundu DJ, Inuganti A, Griss J, Mayer G, Eisenacher M, Pérez E, Uszkoreit J, Pfeuffer J, Sachsenberg T, Yilmaz S, Tiwary S, Cox J, Audain E, Walzer M, Jarnuczak AF, Ternent T, Brazma A, Vizcaíno JA (2019) The PRIDE database and related tools and resources in 2019: improving support for quantification data. Nucleic Acids Res 47(D1):D442-D450 (PubMed ID: 30395289)] with the dataset identifier PXD016836 [https://www.ebi.ac.uk/pride/archive/projects/PXD016836]
The following databases were used in this study:
The Cancer Genome Atlas (TCGA - https://tcga-data.nci.nih.gov/tcga/);
GSE21034 (https://www.ncbi.nlm.nih.gov/geo/query/acc.cgi?acc=GSE21034);
STRING v11.0 (https://string-db.org/cgi/input.pl).
All the data supporting the findings of this study are available within the article and its supplementary information files and from the corresponding author upon reasonable request. A reporting summary for this article is available as Supplementary Information file. The source data underlying Figures 1-7 and Supplementary Figures 1-6 are provided as a Source Data File.

Field-specific reporting

Please select the one below that is the best fit for your research. If you are not sure, read the appropriate sections before making your selection.

☒ Life sciences ☐ Behavioural & social sciences ☐ Ecological, evolutionary & environmental sciences

For a reference copy of the document with all sections, see nature.com/documents/nr-reporting-summary-flat.pdf

Life sciences study design

All studies must disclose on these points even when the disclosure is negative.

Sample size	At least three samples were used per experiment (minimal n = 3). This number is sufficient to support the statistical analyses performed in this manuscript.<br>For mice experiment, a pilot study demonstrated that orthotopic injection of LNCaP AI cells generated tumours in 80% of the cases. Therefore the initial number of mice (14; 7 per group) was determined to ensure at least 5 replicates (tumours) per group and allow to perform statistical analysis.
Data exclusions	No data were excluded from analyses
Replication	Results were replicated at least three times. All attempts at replication were successful.
Randomization	Mice were randomly allocated into experimental groups at the time of the injection.
Blinding	Orthotopic injection experiment was performed by two investigators. One investigator was harvesting and counting the cells before handing the cell suspension to the second investigator, who carried the actual surgery blindly (without knowing which condition was injected).

Reporting for specific materials, systems and methods

We require information from authors about some types of materials, experimental systems and methods used in many studies. Here, indicate whether each material, system or method listed is relevant to your study. If you are not sure if a list item applies to your research, read the appropriate section before selecting a response.

Materials & experimental systems

n/a	Involved in the study	
☐	☒	Antibodies
☐	☒	Eukaryotic cell lines
☒	☐	Palaeontology
☐	☒	Animals and other organisms
☐	☒	Human research participants
☒	☐	Clinical data

Methods

n/a	Involved in the study	
☒	☐	ChIP-seq
☒	☐	Flow cytometry
☒	☐	MRI-based neuroimaging

Antibodies

Antibodies used

PSA sc-7638 Santa Cruz Biotechnology - 1/1000 dilution
AR sc-816 Santa Cruz Biotechnology - 1/1000 dilution
HSC70 sc-7298 Santa Cruz Biotechnology - 1/1000 dilution
FKBP5 #12210 Cell Signaling Technology - 1/1000 dilution
ACC #3676 Cell Signaling Technology - 1/1000 dilution
ACLY #4332 Cell Signaling Technology - 1/1000 dilution
p-ACLY #4331 Cell Signaling Technology - 1/1000 dilution
PKM1/2 #3190 Cell Signaling Technology - 1/1000 dilution
GLUT1 #12939 Cell Signaling Technology - 1/1000 dilution

AMPKa #2603 Cell Signaling Technology - 1/1000 dilution
p-AMPKa #2535 Cell Signaling Technology - 1/1000 dilution
CPT1a #12252 Cell Signaling Technology - 1/1000 dilution
BIP #3177 Cell Signaling Technology - 1/1000 dilution
DNAJC3 #2940 Cell Signaling Technology - 1/1000 dilution
XBP1s #40435 Cell Signaling Technology - 1/1000 dilution
CHOP #2895 Cell Signaling Technology - 1/1000 dilution
EPCAM #93790 Cell Signaling Technology - 1/1000 dilution
ATF4 #11815 Cell Signaling Technology - 1/1000 dilution
PARP #9532 Cell Signaling Technology - 1/1000 dilution
DECR1 ab95965 Abcam - 1/1000 dilution
GPX4 ab125066 Abcam - 1/1000 dilution
AR ChIP grade #5153 Cell Signaling Technology - 1ug/4ug chromatin
AR ChIP grade 17-10489 Millipore - 1ug/4ug chromatin
EnVision detection system K500711-2 Agilent - 1/1 dilution
Goat anti-rabbit Alexa488 ab150077 abcam - 1/3000 dilution
Anti-rabbit IgG HRP-linked #7074 Cell Signaling Technology - 1/3000 dilution
Anti-mouse IgG HRP-linked #7076 Cell Signaling Technology - 1/3000 dilution

Validation

PSA sc-7638 Santa Cruz Biotechnology - validated for WB in βME-treated human prostate tissue extract (scbt website)
AR sc-816 Santa Cruz Biotechnology - validated for WB using untreated and Brefeldin A treated LNCaP whole cell lysates (scbt website)
HSC70 sc-7298 Santa Cruz Biotechnology - validated by WB using K-562, Hep G2, HCT-116, 3T3-L1, MCF7 and C6 whole cell lysates (scbt website).
FKBP5 #12210 Cell Signaling Technology - validated by WB using HT-1080 and Hela cells (CST website)
ACC #3676 Cell Signaling Technology - validated by WB using 3T3, 293 and various cell lines (CST website)
ACLY #4332 Cell Signaling Technology - validated by WB using Hela, 3T3, C6 and COS cells (CST website)
p-ACLY #4331 Cell Signaling Technology - validated by WB using PDGF-treated 3T3 cells (CST website)
PKM1/2 #3190 Cell Signaling Technology - validated by WB using MCF-7, HepG2 and various cell lines (CST website)
GLUT1 #12939 Cell Signaling Technology - validated by WB using RD, Huh6, 3T3 and A204 cells (CST website)
AMPKa #2603 Cell Signaling Technology - validated by WB using 293, COS, PC12 cells and mouse brain extracts (CST website)
p-AMPKa #2535 Cell Signaling Technology - validated by WB using oligomycin-treated C2C12 cells (CST website)
BIP #3177 Cell Signaling Technology - validated by WB using A204, L929, U-87MG (CST website)
DNAJC3 #2940 Cell Signaling Technology - validated by WB using 3T3, Hepal, bTC6 and NIT-1 cells (CST website)
XBP1s #40435 Cell Signaling Technology - validated by WB using tunicamycin-treated 293T cells (CST website)
CHOP #2895 Cell Signaling Technology - validated by WB using thapsigargin treated C6 and A204 cells (CST website)
EPCAM #93790 Cell Signaling Technology - validated by WB using HCT116,HT-29 and various cell lines (CST website)
ATF4 #11815 Cell Signaling Technology - validated by WB using tunicamycin-treated 293 and Hela cells (CST website)
PARP #9532 Cell Signaling Technology - validated by WB using 293 PARP KO cells (CST website)
DECR1 ab95965 Abcam - validated by WB using HepG2 cells (CST website)
GPX4 ab125066 Abcam - validated by WB using mouse and rats testis lysates
AR ChIP grade #5153 Cell Signaling Technology - validated by CHiP on LNCaP cells grown in phenol red free medium and 5% charcoal stripped FBS for 3 d then treated with dihydrotestosterone (DHT, 10 nM) for 4 hours (CST website)
AR ChIP grade 17-10489 Millipore - validated by CHiP on DU145 cells stably transfected with a 7 kD-PSA promoter, different AR poly A lentiviral vectors, and a Flag-tagged hZMIZ1 vector (Millipore website)

All antibodies have been validated for WB on human cell lines in this study (clear band at the right Mw)
AR ChIP antibodies have been validated using CHiP on LNCaP cells (KLK3 as a positive control)

Eukaryotic cell lines

Policy information about cell lines

Cell line source(s)

LNCaP, CWR22Res, VCap and C4-2 cells were purchased from ATCC. Treatment-resistant cell lines were generated by long term cell culture in the presence of the specific inhibitor.

Authentication

All cell lines were authenticated by STR DNA profiling.

Mycoplasma contamination

All cell lines were tested negative for mycoplasma contamination.

Commonly misidentified lines
(See ICLAC register)

The study did not involve misidentified cell lines.

Animals and other organisms

Policy information about studies involving animals; ARRIVE guidelines recommended for reporting animal research

Laboratory animals

male CD1 nude mice; 10 weeks old (at the time of surgery). Mice were ordered from Charles River (U.K), housed in randomised groups of five, at 19°C to 23°C with a 12-hour light-dark cycle, and were fed a conventional diet (Rat and Mouse Standard Expanded, B&K Universal, U. K.) with mains water ad libitum. They were housed in an enriched environment, with igloos, cardboard tubes and chewing sticks.

Wild animals	The study did not involve wild animals.
Field-collected samples	The study did not involve samples collected from the field,
Ethics oversight	In vivo orthograft experiments were performed in accordance with the ARRIVE guidelines, and by a local ethics committee under the Project Licence P5EE22AEE in full compliance with the UK Home Office regulations (UK Animals (Scientific Procedures) Act 1986).

Note that full information on the approval of the study protocol must also be provided in the manuscript.

Human research participants

Policy information about studies involving human research participants

Population characteristics	Tissue microarray performed on human biopsies was used to assess DECR1 expression in matched sample biopsies from particular individuals before and after ADT-deprivation. Patients were all males from 54 to 80 years old (average 69) that were initially diagnosed for prostate cancer and underwent ADT.
Recruitment	Patient recruitment is unlikely to impact the results as we are comparing matched sample biopsies from particular individuals before and after ADT-deprivation
Ethics oversight	This study was approved by the West of Scotland Research Ethics Committee (05/S0704/94). All subjects provided an informed consent and all experiments conformed to the principles set out in the WMA Declaration of Helsinki and the Department of Health and Human Services Belmont Report.

Note that full information on the approval of the study protocol must also be provided in the manuscript.
